# Clinical and epidemiological profiles of six respiratory pathogens in hospitalized children in Lixin County, Anhui, China (2024–2025)

**DOI:** 10.3389/fped.2026.1918213

**Published:** 2026-08-20

**Authors:** Tong Xu, Xiaolei Wu, Fang Lin

**Affiliations:** 1 Pediatric Ward 2, Lixin County People’s Hospital, Anhui, China; 2 Department of Laboratory Medicine, Lixin County People’s Hospital, Anhui, China

**Keywords:** acute respiratory infections (ARI), clinical profiles, epidemiological characteristics, PCR, pediatric, respiratory pathogens

## Abstract

**Background:**

Acute respiratory infections are caused by a diverse range of pathogens. This study aimed to investigate the epidemiological profiles and clinical features of major respiratory pathogens by analyzing their detection rates in hospitalized pediatric patients.

**Methods:**

Nasopharyngeal swab specimens were retrospectively enrolled from pediatric inpatients diagnosed with acute respiratory infections at the Department of Pediatrics, Lixin County People's Hospital, between February 2024 and August 2025. All specimens were subjected to polymerase chain reaction (PCR) assays targeting six major respiratory pathogens, namely adenovirus, respiratory syncytial virus, influenza A virus, influenza B virus, parainfluenza virus, and *Mycoplasma pneumoniae*. Based on the PCR results, we further delineated the epidemiological trends, age-stratified distribution, and corresponding clinical profiles associated with each identified pathogen.

**Results:**

The epidemiological and clinical profiles of the six detected respiratory pathogens are summarized below. In brief, the overall positivity rate of pathogens exhibited winter-spring predominance, with adenovirus and RSV showing the broadest outbreak windows (November–July and November–May, respectively), while influenza A was more restricted (December–March). *Mycoplasma pneumoniae* displayed a sporadic annual pattern without a statistically significant seasonal peak. Age distribution varied substantially: RSV targeted children < 3 years, M. pneumoniae affected those > 1 year, and influenza A showed a slight infant-toddler predominance. Clinically, while fever and cough were universal, RSV was distinguished by a significantly elevated wheezing rate (27.57% vs. 3.97%–9.52% for others), Febrile seizures were most frequently observed in influenza A virus infections (6.29%), followed by adenovirus (3.57%). Dyspnea or cyanosis was uniformly rare across all pathogens (≤ 0.45%).

**Conclusion:**

During the winter season, multiple respiratory pathogens tend to circulate actively. Acute respiratory infections caused by different pathogens exhibit distinct age distributions and clinical manifestations. These features, when combined with local epidemiological data, may assist clinicians in promptly identifying the likely causative pathogen.

## Background

Acute respiratory infections (ARIs) are among the leading causes of pediatric healthcare visits worldwide, especially in children under 5 years of age, and represent a major contributor to both outpatient consultations and hospitalizations, particularly in developing countries. These infections constitute a considerable burden on global public health (1–3). Epidemiological data indicate that young children experience an average of 3–6 respiratory infections annually. Moreover, recurrent episodes have been shown to adversely affect children's daily activities, school performance, and psychological well-being, as well as impose emotional distress on their parents.

ARIs can be attributed to a diverse range of pathogens (4). Viral infections are most common in infants and young children, with rhinovirus, influenza virus, adenovirus, and respiratory syncytial virus (RSV) being the predominant causative agents. In older children, ARIs are more frequently attributed to *Mycoplasma pneumoniae*. The clinical manifestations of ARIs caused by different pathogens are generally similar and commonly include fever, rhinorrhea, cough, expectoration, and wheezing. Severe cases may additionally present with respiratory distress. However, disease course, severity, complication rates, therapeutic approaches, and prognosis differ substantially according to the causative pathogen. Therefore, timely pathogen identification can assist clinicians in formulating more precise treatment regimens, predicting disease trajectory and prognosis, and reducing unnecessary antibiotic use. Furthermore, prompt etiological diagnosis may help alleviate anxiety among pediatric patients and their caregivers.

Respiratory pathogens are primarily transmitted via respiratory droplets, and their prevalence is closely associated with seasonal variations, geographic location, socioeconomic status, population density, and public health interventions. During periods of marked temperature fluctuations or seasonal transitions—particularly in winter and spring—outbreaks of multiple respiratory pathogens are frequently observed. Lixin County is situated in an inland area of central China and has a typical plain terrain, with marked seasonal changes in temperature and weather conditions. The county also features a relatively underdeveloped economy, limited transport infrastructure, high population density, and inadequate sanitation facilities. Collectively, these factors may facilitate the transmission of respiratory pathogens. Consequently, the epidemiological patterns of respiratory pathogens in this region may differ considerably from those observed in other geographic settings.

Different pathogens exhibit distinct seasonal patterns; for example, influenza viruses are most prevalent in autumn and winter (5), while *Mycoplasma pneumoniae* infections peak in autumn (6). However, the stringent COVID-19 containment measures implemented in China between 2019 and 2021 significantly reduced pathogen transmission, leading to a marked decline in ARIs (7, 8). The subsequent “immunological debt” (9)—attributed to prolonged reduced exposure to diverse pathogens in children—contributed to a substantial resurgence of respiratory infections following the relaxation of control measures in 2022. For instance, a persistent Mycoplasma pneumoniae outbreak occurred in 2023 (10), which deviated markedly from historical epidemiological patterns. Therefore, the present study aimed to investigate the epidemiological trends and clinical characteristics of respiratory pathogens causing ARIs in Lixin County between 2024 and 2025—findings that hold significant implications for local disease prevention and control strategies.

## Methods

### Study participants

This retrospective study included children who were admitted to the Department of Pediatrics at Lixin People's Hospital between February 2024 and August 2025 with a diagnosis of acute respiratory infection (ARI). The primary clinical manifestations included fever, cough, and wheezing, while severe cases presented with signs of respiratory failure, such as tachypnea or lip cyanosis. The inclusion criteria were as follows (1): age < 15 years; (2) presence of at least one respiratory symptom (e.g., fever, cough, or wheezing) and a clinical diagnosis of bronchitis, pneumonia, or febrile infectious illness. The exclusion criteria were: (1) diagnoses other than respiratory tract infections; (2) nosocomial respiratory infections. The study protocol was approved by the Ethics Committee of Lixin County People's Hospital (approval no. LXXRMYY-2026LW004), which waived the requirement for written informed consent due to the retrospective nature of the study.

### Specimen collection and pathogen nucleic acid testing

Nasopharyngeal swabs were collected from all patients who met the eligibility criteria. All specimen collection personnel had undergone standardized training and obtained certification prior to performing nasopharyngeal swab sampling. Immediately after collection, specimens were placed in collection tubes containing viral transport medium and were promptly transported to the hospital's clinical laboratory. Subsequently, all specimens were tested for respiratory pathogens using polymerase chain reaction (PCR) in accordance with the manufacturer's instructions (6-Item Respiratory Pathogen Detection Kit; Beijing Zhuocheng Huisheng Biotechnology Co., Ltd.). This assay was designed for the *in vitro* qualitative detection of nucleic acids from six respiratory pathogens in throat swabs, sputum, and bronchoalveolar lavage fluid (BALF). The target pathogens comprised *Mycoplasma pneumoniae*, adenovirus (groups B, C, and E), influenza A virus [subtypes H1N1, H3N2, H1N1 (2009), H5N1, and H7N9], influenza B virus (Victoria and Yamagata lineages), parainfluenza virus (types 1–4), and RSV (subtypes A and B). This kit did not distinguish between subtypes/Lineages within each pathogen category.

### Data collection, consolidation, and analysis

The test results were stratified by gender and age group, with the latter categorized as infants (< 1 year), toddlers (1–3 years), preschoolers (4–6 years), and school-aged children (7–15 years). In addition, all results were analyzed according to the month of admission. The clinical characteristics associated with each pathogen were also described. All statistical analyses were performed using IBM SPSS Statistics (version 27, IBM Corp., Armonk, NY, USA), and graphical representations were generated using GraphPad Prism (version 10, GraphPad Software, Boston, MA, USA). Categorical variables were presented as frequencies and percentages. Between-group comparisons were performed using the Chi-Square (*χ*^2^) test, and a two-sided *P* value < 0.05 was considered statistically significant. Hypothesis testing was precluded in groups with small sample sizes, for which only descriptive data were presented.

## Result

### Demographic characteristics

From February 2024 to August 2025, a total of 3,955 specimens were collected, of which 2,413 (61.01%) were from males and 1,542 (38.99%) from females. Among these, 752 male specimens (31.16%) and 503 female specimens (32.62%) tested positive for respiratory pathogens. The pathogen positivity rate did not differ significantly between males and females (*χ*^2^ = 0.920, *P* = 0.337). The positivity rates in each age group were as follows: infants, 315/1,112 (28.33%); toddlers, 433/1,440 (30.07%); preschoolers, 337/833 (40.46%); and school-aged children, 170/570 (29.82%). The positivity rates varied significantly across age groups (*χ*^2^ = 38.012, *P* < 0.001), with higher rates observed in infants and toddlers than in older children. The demographic characteristics and detailed pathogen detection results are presented in Tables 1, 2, respectively.

**Table 1:** Demographic characteristics of 3,955 hospitalized children with acute respiratory infections in Lixin County, Anhui, China (February 2024 – August 2025), stratified by sex and age group.

	Total n(%)	positive cases n(%)
Gender		
Male	2,413 (61.01%)	752 (31.16%)
Female	1,542 (38.99%)	503 (32.62%)
Age groups		
under 1 year	1,112 (28.12%)	315 (28.33%)
1–3 years	1,440 (36.41%)	433 (30.07%)
4–6 years	833 (21.06%)	337 (40.46%)
7–15 years	570 (14.41%)	170 (29.82%)

**Table 2:** Age-group distribution of six respiratory pathogens (adenovirus, influenza A virus, influenza B virus, respiratory syncytial virus, parainfluenza virus, and Mycoplasma pneumoniae) among 1,255 PCR-positive hospitalized children in Lixin County, Anhui, China.

Age groups	ADV *n*(%)	IFA *n*(%)	IFB *n*(%)	RSV *n*(%)	HPIV *n*(%)	MP *n*(%)	Total *n*(%)
under 1 year	61 (13.62%)	80 (26.49%)	11 (32.35%)	145 (53.31%)	4 (40.00%)	14 (7.41%)	315
1–3 years	128 (28.57%)	120 (39.74%)	15 (44.12%)	102 (37.50%)	5 (50.00%)	63 (33.33%)	433
4–6 years	193 (43.08%)	62 (20.53%)	6 (17.65%)	16 (5.88%)	1 (10.00%)	59 (31.22%)	337
7–15 years	66 (14.73%)	40 (13.25%)	2 (5.88%)	9 (3.31%)	0 (0.00%)	53 (28.04%)	170
Total	448	302	34	272	10	189	1,255
*χ* ^2^	162.638	1.654	3.165	122.968	-^1^	66.095	38.012
*P*-VALUE	<0.001	0.647	0.367	<0.001	-^2^	<0.001	<0.001

ADV, adenovirus; IFA, influenza A virus; IFB, influenza B virus; RSV, respiratory syncytial virus; HPIV, human parainfluenza virus; MP, Mycoplasma pneumoniae; *χ*², Chi-square test statistic.

1–2 : Given the limited sample size of the HPIV group, the chi-square test was deemed inappropriate for hypothesis testing. Accordingly, the analyses were confined to descriptive statistics only.

### Pathogen detection rates and epidemiological trends

**Figure 1:**
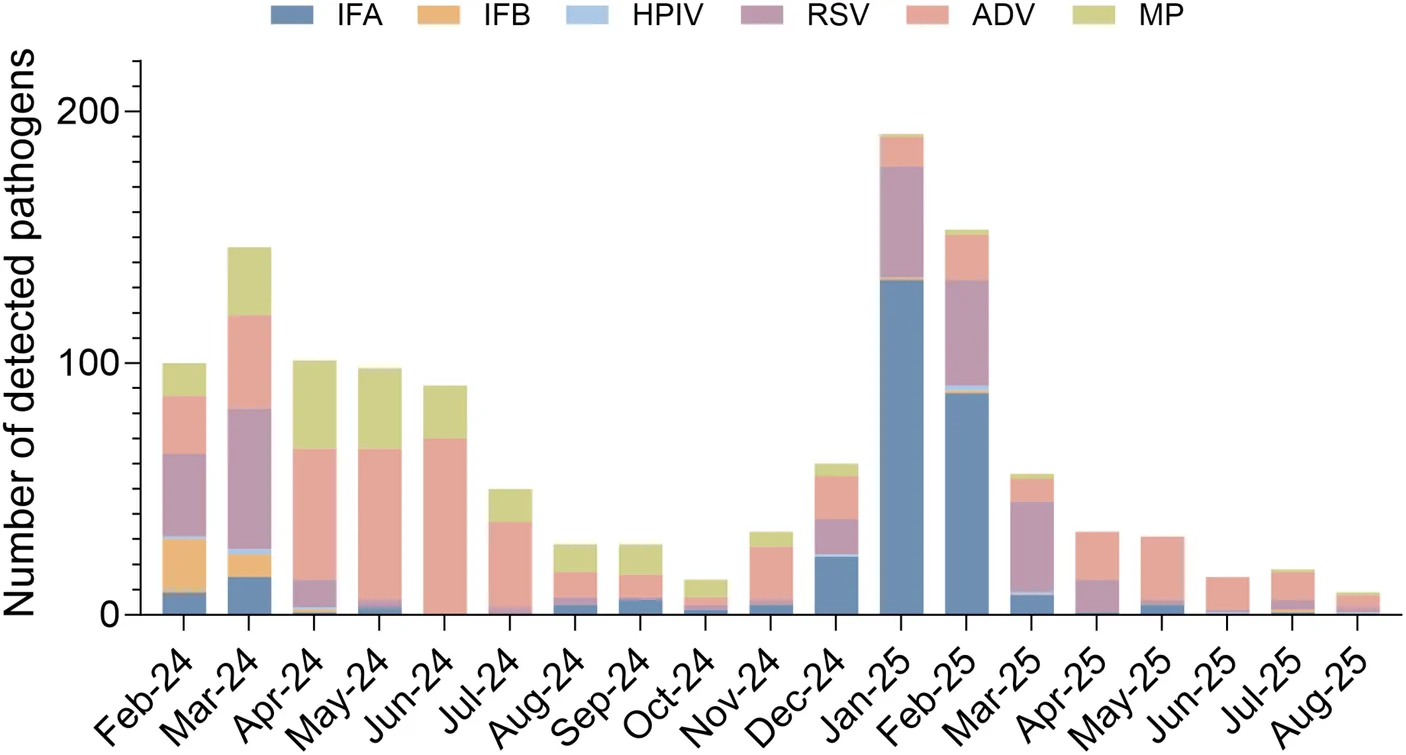
Monthly prevalence trends of adenovirus, influenza A virus, influenza B virus, RSV, parainfluenza virus, and Mycoplasma pneumoniae in hospitalized children with acute respiratory infections in Lixin County, Anhui, China (February 2024 – August 2025). ADV, adenovirus; IFA, influenza A virus; IFB, influenza B virus; RSV, respiratory syncytial virus; HPIV, human parainfluenza virus; MP, Mycoplasma pneumoniae.

**Figure 2:**
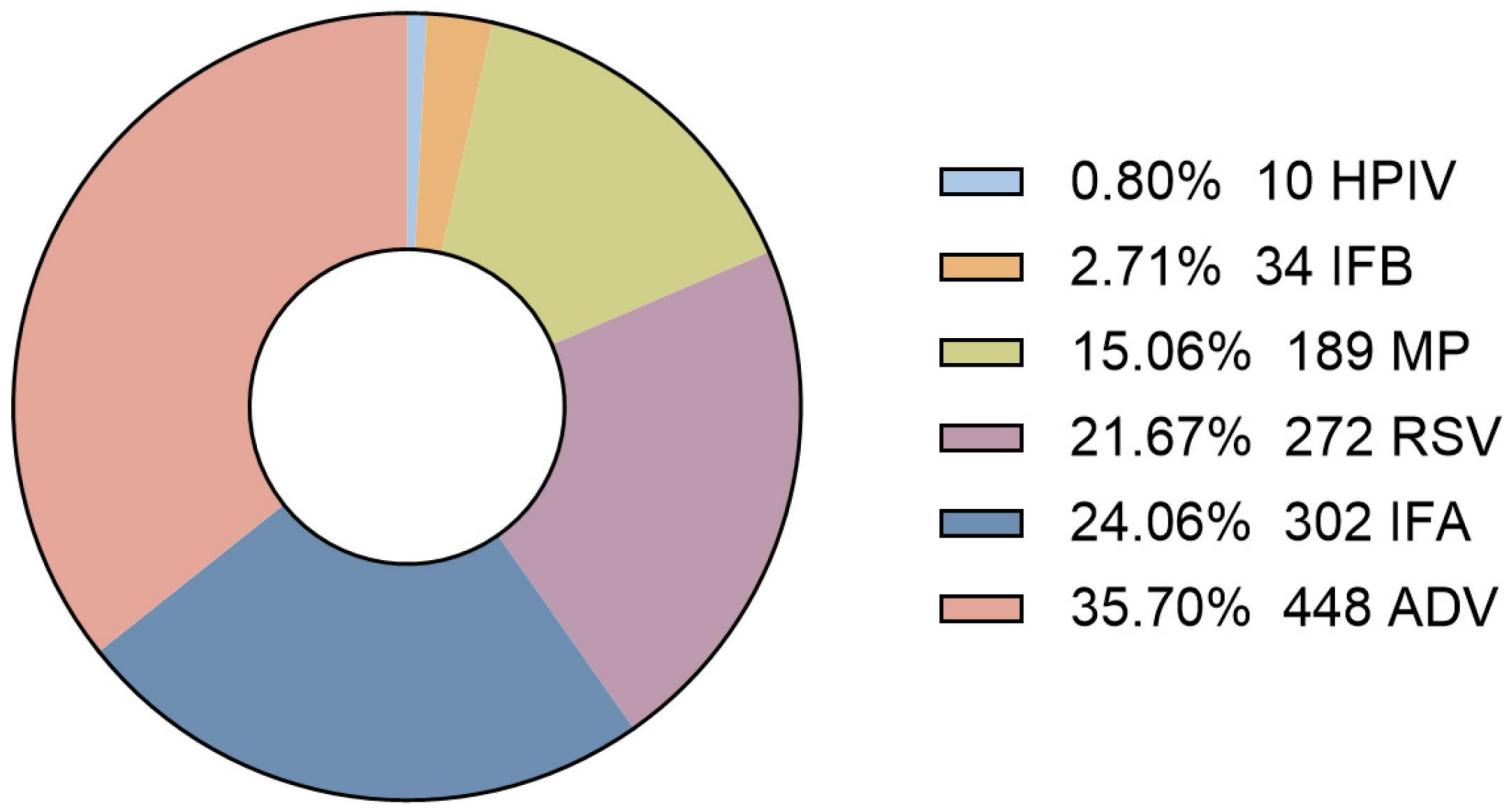
Overall detection rates of six respiratory pathogens (adenovirus, influenza A, influenza B, RSV, parainfluenza, and M. pneumoniae) among 1,255 positive specimens from 3,955 hospitalized pediatric patients with acute respiratory infections in Lixin County, Anhui, China (February 2024 – August 2025). ADV, adenovirus; IFA, influenza A virus; IFB, influenza B virus; RSV, respiratory syncytial virus; HPIV, human parainfluenza virus; MP, Mycoplasma pneumoniae.

The overall peak of ARIs occurred in May 2024, predominantly attributed to concurrent adenovirus and *Mycoplasma pneumoniae* infections, whereas the peak in January 2025 was primarily driven by influenza A virus. Influenza A outbreaks occurred from February to April 2024 and again from November 2024 to March 2025, exhibiting similar seasonal patterns across the two epidemic years. Adenovirus exhibited a high incidence from February to July 2024, whereas in 2025, cases occurred sporadically throughout the year, with a marked decrease in incidence compared with the previous year and no evidence of widespread outbreaks. *Mycoplasma pneumoniae* showed an epidemiological trend similar to that of adenovirus, with high incidence before July 2024; however, no widespread outbreaks were observed from that time until August 2025. RSV caused outbreaks from February to April 2024 and again from November 2024 to April 2025, exhibiting similar seasonal patterns in both epidemic years. Parainfluenza virus and influenza B virus were limited to sporadic cases throughout the two-year study period, with no significant epidemic trends observed. Overall, the detection rates of influenza A virus, influenza B virus, and adenovirus were significantly higher than those of other pathogens. Detailed detection data and the corresponding epidemiological trends for each pathogen are presented in Table 3 and Figures 1,2, respectively.

**Table 3:** Monthly distribution of PCR-positive cases for six respiratory pathogens among hospitalized children in Lixin County, Anhui, China, from February 2024 to August 2025 (including monthly total specimens and negative cases).

Date	IFA	IFB	HPIV	RSV	ADV	MP	Negative	Total
Feb-24	9	21	1	33	23	13	95	195
Mar-24	15	9	2	56	37	27	125	271
Apr-24	1	1	1	11	52	35	166	267
May-24	3	0	0	3	60	32	208	306
Jun-24	0	0	0	0	70	21	196	287
Jul-24	0	0	0	3	34	13	142	192
Aug-24	4	0	0	3	10	11	120	148
Sep-24	6	0	0	1	9	12	142	170
Oct-24	2	0	0	2	3	7	154	168
Nov-24	4	0	0	2	21	6	169	202
Dec-24	23	0	1	14	17	5	195	255
Jan-25	133	1	0	44	12	1	141	332
Feb-25	88	1	2	42	18	2	106	259
Mar-25	8	0	1	36	9	2	113	169
Apr-25	1	0	0	13	19	0	126	159
May-25	4	0	0	2	25	0	162	193
Jun-25	0	0	1	1	13	0	139	154
Jul-25	1	1	0	4	11	1	111	129
Aug-25	0	0	1	2	5	1	90	99
Total	302	34	10	272	448	189	2,700	3,955

ADV, adenovirus; IFA, influenza A virus; IFB, influenza B virus; HPIV, human parainfluenza virus; RSV, respiratory syncytial virus; MP, Mycoplasma pneumoniae.

### Analysis of clinical features

The clinical manifestations of ARIs share common features across different pathogens, yet certain symptom profiles vary by etiological agent. Hospitalized patients most commonly presented with fever, cough, and wheezing, while severe cases exhibited dyspnea or cyanosis; a subset of children were admitted due to febrile seizures. Fever was more prominent following adenovirus and influenza virus infections (*χ*^2^ = 106.084, *P* < 0.001). In addition, febrile seizures occurred more frequently in these groups (*χ*^2^ = 17.893, *P* = 0.003). RSV infection was significantly more likely to cause wheezing compared with other pathogens (*χ*^2^ = 92.198, *P* < 0.001). Severe RSV cases also presented with dyspnea and cyanosis. In contrast, parainfluenza virus and influenza B virus infections were associated with lower incidence and fewer specific clinical features during the study period. Detailed clinical manifestations associated with each pathogen are presented in Table 4.

**Table 4:** Comparison of clinical features (fever, cough, wheezing, dyspnea/cyanosis, and febrile seizures) among hospitalized children infected with adenovirus, influenza A, influenza B, RSV, parainfluenza virus, and Mycoplasma pneumoniae in Lixin County, Anhui, China.

Clinical features	ADV *n*(%)	IFA *n*(%)	IFB *n*(%)	RSV *n*(%)	HPIV *n*(%)	MP *n*(%)	χ^2^	*P*-value
Fever	364 (81.25%)	253 (83.77%)	30 (88.24%)	136 (50.00%)	6 (60.00%)	139 (73.54%)	106.084	<0.001
cough	195 (43.53%)	142 (47.02%)	30 (88.24%)	247 (90.81%)	8 (80.00%)	175 (92.59%)	314.540	<0.001
wheezing	26 (5.80%)	12 (3.97%)	3 (8.82%)	75 (27.57%)	2 (20.00%)	18 (9.52%)	92.198	<0.001
Dyspnea or cyanosis	2 (0.45%)	1 (0.33%)	0 (0.00%)	6 (2.21%)	0 (0.00%)	0 (0.00%)	10.123	0.072
Seizures	16 (3.57%)	19 (6.29%)	0 (0.00%)	6 (2.21%)	0 (0.00%)	1 (0.53%)	17.893	0.003

ADV, adenovirus; IFA, influenza A virus; IFB, influenza B virus; RSV, respiratory syncytial virus; HPIV, human parainfluenza virus; MP, Mycoplasma pneumoniae; *χ*², Chi-square test statistic; *P*-value, probability value.

## Discussion

Respiratory infections are characterized by acute onset in most patients, with clinical manifestations including fever, rhinorrhea, cough, wheezing, and dyspnea. Although a wide variety of pathogens can cause ARIs in children (11, 12), the present study targeted six specific respiratory pathogens—RSV, adenovirus, influenza A virus, influenza B virus, *Mycoplasma pneumoniae*, and parainfluenza virus—because they are generally associated with more severe clinical manifestations, higher complication rates, and greater mortality compared with other respiratory viruses such as rhinoviruses. For instance, the incidence of severe complications—including pneumonia, respiratory failure, myocarditis, and acute necrotizing encephalopathy—is significantly higher in influenza virus infections than in rhinovirus infections. This distinction is particularly noteworthy given that the initial symptoms of these two viral infections are often clinically indistinguishable.

Adenovirus comprises 7 species (subgroups) and 67 serotypes. The serotypes most commonly associated with respiratory infections in humans include types 3, 7, 11, 14, 21, and 55 (13). Clinical manifestations vary by serotype. Mild cases typically present with upper respiratory tract symptoms, including fever, rhinorrhea, and sore throat, whereas more severe cases may progress to pharyngoconjunctival fever, characterized by conjunctivitis, pharyngeal exudates, and persistent high fever. Certain serotypes can cause severe pneumonia, acute respiratory distress syndrome, respiratory failure, heart failure, and toxic encephalopathy. In later stages, infection may also lead to obstructive bronchiolitis, which can substantially impair quality of life (14).

Most previous studies have reported that adenovirus infections peak during winter and spring (15), with the majority of cases occurring in children under 3 years of age. In contrast to these findings, the present study revealed a gradual increase in adenovirus cases during the first half of 2024 (February to June), followed by a rapid decline after August. In 2025, only sporadic cases were observed throughout the year, with no clear winter or spring outbreaks. The incidence was predominantly concentrated in children aged 1–6 years, whereas lower rates were observed in other age groups. Specifically, the age distribution of confirmed adenovirus cases was as follows: infants (< 1 year), 61 (13.62%); toddlers (1–3 years), 128 (28.57%); preschoolers (4–6 years), 193 (43.08%); and school-aged children (7–15 years), 66 (14.73%) (*χ*^2^ = 162.638,*P* < 0.001). The low detection rate in younger children may be partly related to maternal adenovirus antibodies, but this requires further serological investigation.

The primary clinical features of adenovirus infection in this cohort were fever and cough, with some cases presenting with persistent high fever and occasional febrile seizures. Wheezing was observed in severe cases, but severe complications—including severe pneumonia, encephalitis, and septic shock—were rarely documented. It is possible that only specific serotypes are associated with such severe manifestations, and the serotypes circulating in this study population may not have included those highly pathogenic variants. Currently, no specific antiviral therapy is available for adenovirus infections; management remains largely supportive, including antipyretic treatment and fluid resuscitation.

Influenza is an acute respiratory infectious disease caused by influenza viruses and circulates worldwide, with seasonal peaks typically occurring in winter and spring. Influenza viruses belong to the family Orthomyxoviridae and are classified into types A, B, and C based on their matrix and nucleoprotein characteristics (16). In the present study, influenza A virus remained prevalent during the winter and spring months (December to March) of the 2024–2025 season, which is consistent with previously reported epidemiological patterns (17).

Although the general population is susceptible to influenza, the age distribution of hospitalized influenza A cases in this study was as follows: infants (< 1 year), 80 (26.49%); toddlers (1–3 years), 120 (39.74%); preschoolers (4–6 years), 62 (20.53%); and school-aged children (7–15 years), 40 (13.25%) (*χ*^2^ = 1.654,*P* = 0.647), indicating no statistically significant difference in hospitalization rates across age groups.

Similar to adenovirus infection, the primary clinical manifestations of influenza A in this cohort were fever and cough. Persistent high fever occasionally led to febrile seizures, whereas wheezing was rarely observed, likely due to distinct pathogenic mechanisms. Fulminant myocarditis and necrotizing encephalopathy are among the most severe complications of influenza and can be rapidly fatal (18, 19); however, their incidence is low, and none of the 302 patients with influenza A in this study developed these conditions.

Specific antiviral agents, including oseltamivir and baloxavir marboxil, are available for influenza (20). Early administration—ideally within 24 h of symptom onset—has been shown to significantly reduce the incidence of complications in patients with clinically diagnosed influenza. Influenza B virus had a low incidence throughout the study period and occasionally co-circulated with influenza A during winter and spring, but did not cause widespread outbreaks.

Parainfluenza viruses belong to the family Paramyxoviridae and are classified into four serotypes (types 1–4) (21). Epidemiological studies have shown that HPIVs exhibit seasonal variation: HPIV-3 is most prevalent in winter and spring, HPIV-1 in summer, and HPIV-4 in winter, whereas HPIV-2 is detected at low levels throughout the year without a clear seasonal pattern. In the present study, HPIV infections occurred sporadically throughout 2024 and 2025 and remained uncommon. This may be attributed to the relatively lower virulence and transmissibility of HPIVs compared with influenza virus, adenovirus, and RSV.

RSV is one of the major pathogens responsible for ARIs in infants and young children. It is highly contagious, causes substantial morbidity, and can cause widespread outbreaks (22). In line with this, the present study found that large-scale RSV outbreaks occurred during winter and spring, specifically from February to April 2024 and from December 2024 to April 2025. Hospitalized children with RSV infection were predominantly under 3 years of age. The age distribution of RSV-positive cases was as follows: infants (< 1 year), 145 (53.31%); toddlers (1–3 years), 102 (37.50%); preschoolers (4–6 years), 16 (5.88%); and school-aged children (7–15 years), 9 (3.31%) (*χ*^2^ = 122.968,*P* < 0.001). In addition to fever and cough, RSV was significantly more likely to cause wheezing than other respiratory pathogens (27.57%, *χ*^2^ = 92.198,*P* < 0.001). Severe cases presented with paroxysmal cough, dyspnea, and cyanosis, and some progressed to respiratory failure or acute toxic encephalopathy (23).

Currently, no specific antiviral therapy is available for RSV, and clinical management remains largely supportive, including respiratory support (24); corticosteroids and bronchodilators may be considered for patients with severe wheezing, although their efficacy remains debated. Overall, the prognosis for hospitalized children with RSV pneumonia is generally favorable; however, virus-induced airway injury may lead to recurrent wheezing and increase the future risk of asthma (25).

*Mycoplasma pneumoniae* is one of the major pathogens responsible for community-acquired pneumonia in preschool and school-aged children (26). It can cause bronchopneumonia and lobar pneumonia, and in severe cases, may lead to pulmonary consolidation, atelectasis, and plastic bronchitis. Moreover, M. pneumoniae shows high-level resistance to macrolide antibiotics, which complicates clinical management (27). In China, a large-scale M. pneumoniae outbreak occurred in 2023, and high incidence persisted throughout 2024, before declining markedly by 2025; however, no clear seasonal pattern was observed during this period. This apparent absence of seasonality may be partly attributable to population-level immunity acquired after the widespread 2023–2024 outbreak, although this hypothesis warrants further investigation.

M. pneumoniae infection was significantly associated with age. The age distribution of M. pneumoniae-positive cases was as follows: infants (< 1 year), 14 (7.41%); toddlers (1–3 years), 63 (33.33%); preschoolers (4–6 years), 59 (31.22%); and school-aged children (7–15 years), 53 (28.04%) (*χ*^2^ = 66.095,*P* < 0.001). A possible explanation for this age distribution is that M. pneumoniae primarily induces lung injury through host immune responses, which may be more robust in older children. In addition, maternal antibodies may provide partial protection to infants and young children. This is consistent with previous reports suggesting that the incidence and severity of M. pneumoniae infection increase with age (28).

The predominant clinical manifestations of M. pneumoniae infection in this cohort were fever and cough; wheezing was also observed in some cases. Although pulmonary consolidation and atelectasis were observed in some cases, severe pneumonia manifestations such as dyspnea and cyanosis were not documented in this cohort.

Recent reports have indicated a high rate of macrolide resistance in M. pneumoniae; consequently, fluoroquinolones or tetracyclines may be considered for clinical treatment (29), although close monitoring for adverse reactions is recommended. In addition, corticosteroid therapy may be considered for severe cases, a practice that is supported by the immune-mediated pathogenesis of M. pneumoniae pneumonia (30).

Several limitations of this study should be acknowledged. This study included only hospitalized pediatric patients, and data from those with milder illness who did not require hospitalization were not collected, which may have introduced selection bias. Consequently, our findings may not be fully representative of the broader pediatric population with ARIs in the community. Pathogen detection was performed using PCR-based nucleic acid amplification. Test results may have been affected by variations in specimen collection quality, contamination during sampling, and patient compliance, potentially leading to false-negative results. This may have led to an underestimation of the prevalence of certain pathogens. In future studies, combining PCR with serological assays and enrolling a larger sample size would help enhance the reliability of the findings.

## Conclusion

This study provides evidence that outbreaks of respiratory infections in children predominantly occur during the winter season, with epidemiological patterns largely mirroring those observed prior to the COVID-19 pandemic. These findings suggest that early vaccination and non-pharmacological preventive measures may be considered to reduce the incidence of ARIs.

Although the clinical manifestations of ARIs share common features, certain pathogen-specific differences may aid in differential diagnosis. For instance, infants and young children presenting with persistent high fever without wheezing may have adenovirus or influenza virus infections. In such cases, early oseltamivir therapy may be considered, particularly when local epidemiological data suggest influenza activity. Infants and young children with wheezing accompanied by high fever may have adenovirus infection, which tends to have a prolonged course, higher complication rates, and greater management challenges. Wheezing without high fever in infants and young children is more likely attributable to RSV infection, for which management is primarily supportive, including respiratory support.

*Mycoplasma pneumoniae* infection was uncommon in infants under 1 year of age in this cohort; therefore, macrolides may not be preferred as first-line antibiotics in this age group. In contrast, clinicians should remain vigilant for M. pneumoniae infection in older children presenting with wheezing. In older children, high fever was most commonly associated with influenza virus and adenovirus infections, especially in cases accompanied by febrile seizures.

An early presumptive etiological diagnosis can be made by integrating local epidemiological surveillance data with the child's clinical presentation. This approach may facilitate timely therapeutic decisions and guide targeted public health prevention strategies.

## Data Availability

The raw data supporting the conclusions of this article will be made available by the authors, without undue reservation.
